# Reducing academic cheating through growth mindset: an intervention study and a mechanism analysis

**DOI:** 10.3389/fpsyg.2026.1684354

**Published:** 2026-02-18

**Authors:** Song Chang, Yinghua Bao, Chengyou Zhang, Min Xu, Yunyun Huang, Sufei Xin

**Affiliations:** 1 College of Education, Ludong University, Yantai, China; 2 Collaborative Innovation Center for the Mental Health of Youth from the Era of Conversion of New and Old Kinetic Energy along the Yellow River Basin, Yantai, China; 3 Yantai University, Yantai, China

**Keywords:** academic cheating, growth mindset, intervention, mindset meaning system, university students

## Abstract

Academic dishonesty remains a persistent challenge in higher education, highlighting the need for scalable and cost-effective interventions that target internal motivation. Building on mindset theory, the present research tests the impact of a brief growth-mindset intervention on exam cheating and examines the mindset meaning system as a hypothesized mechanism. Growth mindset refers to beliefs about the malleability of one’s abilities, whereas the mindset meaning system refers to the broader system of meanings organized by individuals’ mindsets, shaping how they interpret effort, performance, and failure in achievement contexts. Study 1 employed a randomized controlled trial (*N* = 120) to evaluate the effect of a single-session growth mindset intervention on cheating behavior during a subsequent test. Study 2 used a cross-sectional survey (*N* = 475) to assess whether the growth mindset indirectly relates to cheating through the mindset meaning system. Results from Study 1 showed that the intervention significantly enhanced growth mindset levels and reduced cheating behavior compared to the control group. In Study 2, analysis of indirect effects revealed that growth mindset was not directly associated with cheating but had an indirect effect via the mindset meaning system. These findings suggest that promoting a growth mindset may serve as a promising strategy for reducing academic cheating.

## 1 Introduction

Academic cheating continues to be a common and intractable problem in higher education today (Rettinger et al., 2024; Zhao et al., 2024b). Although researchers have gained a relatively clear understanding of the factors influencing academic cheating, intervention efforts have yielded limited success, underscoring the need for scalable, cost-effective approaches that target internal motivational processes. Growth mindset—the belief that intelligence and abilities can be developed rather than fixed—provides a theoretically grounded avenue for such interventions (Yeager and Dweck, 2012; Dweck, 2013). However, causal evidence for its impact on academic cheating remains scarce. To address this gap, the present research combines a randomized controlled experiment with a mechanism-focused study to test whether a brief growth-mindset intervention reduces cheating and to explore its underlying mechanisms.

Academic cheating among university students is driven by both contextual and individual factors. Contextual factors include institutional policies such as honor codes (McCabe et al., 1999; Zhao et al., 2023), instructor-related factors such as perceived injustice (Laeeque and Saeed, 2023), and peer influence (Ghanem and Mozahem, 2019; Zhao et al., 2022). At the individual level, relevant factors include personality traits (e.g., impulsivity, conscientiousness, agreeableness) (Anderman et al., 2010; McTernan et al., 2013), academic motivation (Daumiller and Janke, 2020; Krou et al., 2021), moral reasoning (Grym and Liljander, 2016), and demographic variables (e.g., gender, age, major) (Williams et al., 2010; Hensley et al., 2013; Michou et al., 2014; Zhang et al., 2018). However, many of these factors, particularly personality traits and demographic characteristics, are relatively stable and resistant to intervention or change. In contrast, academic motivation variables are more malleable, offering opportunities for effective interventions to reduce academic dishonesty (Duckworth and Yeager, 2015; Krou et al., 2021).

Murdock and Anderman (2006) proposed a theoretical framework to explain how motivational variables influence academic dishonesty. The model revolves around three motivational questions: “What is my purpose?,” “Can I do this?,” and “What are the costs?.” Current research on motivational variables and academic dishonesty primarily focuses on the motivational variables related to the first two questions. When answering “What is my goal?,” the key motivational variable is achievement goals. Numerous empirical studies indicate that students with a performance-oriented goal tend to cheat more than those with a mastery-oriented goal (Anderman and Midgley, 2004; Daumiller and Janke, 2020; Xu et al., 2023). Mastery-oriented students focus on mastering knowledge and value the learning process over outcomes, while performance-oriented students aim to surpass their peers, placing greater emphasis on outcomes, which makes them more prone to cheating. When addressing “Can I achieve my goal?,” self-efficacy and locus of control are considered central motivational constructs. Specifically, students with higher self-efficacy are more confident in their academic abilities and are less likely to cheat compared to those with lower self-efficacy (Murdock et al., 2001; Nora and Zhang, 2010; Tas and Tekkaya, 2010). As for locus of control, students with an internal locus of control are more likely to attribute success and failure to their own efforts, adjusting their level of effort to control future outcomes rather than resorting to academic dishonesty (Perry et al., 2001). Furthermore, when students believe that success and failure are determined by external forces beyond their control, academic dishonesty may be perceived as a way to counteract these external factors and achieve their desired results (Rinn et al., 2014; Krou et al., 2021).

Despite robust correlational evidence linking motivational variables to academic dishonesty, their application in educational interventions has been limited. One challenge is scalability: while some academic motivation variables have shown effective interventions in laboratory settings, they lack effective short-term intervention strategies in educational practice (Daumiller and Janke, 2020). Another challenge is that single-variable interventions may have limited predictive and practical impact; meta-analytic evidence suggests that individual motivational constructs typically account for small-to-moderate proportions of variance in cheating behavior (Krou et al., 2021). These limitations underscore the need for interventions that can simultaneously influence multiple motivational drivers of academic integrity while remaining scalable in real-world educational contexts.

The need for scalable interventions that can influence multiple motivational determinants of cheating aligns closely with the theoretical and practical strengths of the growth mindset framework. A growth mindset serves not only as a belief about malleability but also as the organizing principle of a broader mindset meaning system. This system integrates achievement goals, beliefs about effort, and attributional styles into a coherent motivational structure (Yeager and Dweck, 2020). Individuals with a growth mindset are more likely to adopt mastery-oriented goals, hold positive beliefs about the value of effort, and attribute failures to insufficient effort or ineffective strategies rather than personal limitations (Hong et al., 1999; Blackwell et al., 2007). Conversely, those with a fixed mindset are more likely to adopt performance-oriented goals, hold negative beliefs about effort, and make helpless attributions when facing failure. Thus, the mindset meaning system can be defined as a coherent system of meanings organized by individuals’ mindsets that shapes how they interpret effort, performance, and failure in achievement contexts.

It is noteworthy that the components of the mindset meaning system substantially overlap with those described in the motivational model of academic dishonesty proposed by Murdock and Anderman (2006). Specifically, goal orientation corresponds to the “What is my purpose?” dimension, and beliefs about effort and attributional styles correspond to the “Can I do this?” dimension. This theoretical alignment suggests that fostering a growth mindset could concurrently shift multiple proximal motivational factors that predispose students to cheating. Thus, a key theoretical strength of the mindset framework lies in its capacity to form a meaning system that simultaneously shapes multiple core motivational variables relevant to academic integrity. While prior cross-sectional studies have found that growth mindset is negatively associated with academic dishonesty (Thomas, 2017; Herdian and Rahayu, 2022), causal evidence from randomized controlled trials remains scarce. Moreover, little is known about whether mindset, as the core or source of the meaning system, influences cheating behavior indirectly through the mindset meaning system.

Growth mindset interventions have demonstrated high feasibility and cost-effectiveness in practice. A large-scale study by Yeager et al. (2019) showed that less than 1 hour of online growth mindset guidance can significantly enhance students’ growth mindset and lead to sustained improvements in subsequent academic performance. Additional studies have further confirmed that even brief growth mindset interventions can yield stable effects (Schleider et al., 2019; Burnette et al., 2023; Yu et al., 2024), making growth mindset-based interventions for academic dishonesty both practical and low-cost in the higher education context.

Thus, the overarching aim of this research was to evaluate whether a growth mindset-based intervention could serve as an effective and practical strategy for reducing academic cheating in higher education, and to further explore the underlying motivational mechanism. Study 1 used a randomized controlled trial to test whether a growth mindset intervention could decrease students’ cheating behavior. Study 2 then examined the proposed psychological mechanism, testing whether growth mindset is indirectly related to exam cheating through the mindset meaning system. Based on the theoretical framework and prior empirical evidence, we formulated two hypotheses:

*H1*: A growth mindset intervention will reduce exam cheating behavior among university students.

*H2*: Growth mindset will be indirectly associated with exam cheating through the mindset meaning system, such that stronger growth mindset predicts lower cheating via more adaptive motivational beliefs.

## 2 Study 1

Study 1 employed a randomized controlled experiment to intervene with university students’ growth mindset and examine its effect on reducing exam cheating. Cheating behavior was objectively assessed through experimental methods.

### 2.1 Materials and methods

#### 2.1.1 Participants

A power analysis using G*Power (Faul et al., 2009) suggested that a total sample size of *N* = 128 (64 per condition) would provide 80% power to detect a medium effect (*d* = 0.50) with *α* = 0.05 (two-tailed). A recruitment notice was posted at a public university in Shandong Province, eastern China, and 120 university students enrolled in the experiment. Due to practical constraints, this sample size was slightly below the target. It is nevertheless comparable to prior brief, single-session intervention studies (e.g., *N* = 96 in Schleider and Weisz, 2016, and *N* = 104 in Ball et al., 2025). We randomly assigned them to the intervention (*n* = 60) or control group (*n* = 60). At baseline, no significant differences were found between students in the control and intervention groups in gender (*χ*^2^ = 0.85, *p* = 0.36), age (*t (118)* = 0.25, *p* = 0.80), and grade (*χ*^2^ = 0.44, *p* = 0.93) (see Table 1).

**Table 1:** General characteristics of the participants (*N* = 120).

Characteristic	Classification	Intervention group (*n* = 60)	Control group (*n* = 60)	*p* value
Gender	Male	23	28	0.36
	Female	37	32	
Age	18	12	10	0.80
	19	23	25	
	20	14	15	
	≥21	11	10	
Grade	1	26	27	0.93
	2	17	19	
	3	13	11	
	4	4	3	

#### 2.1.2 Procedure

Sessions were run in six cohorts (20 participants per session). All experimental sessions were conducted in the same classroom and scheduled within a consistent evening time window (4:00 p.m. to 9:00 p.m.). For scheduling purposes, sessions were scheduled in a 90-min block (the maximum time window allocated for each cohort), and the procedure was designed to be completed within this window. Upon arrival, participants received standardized instructions and provided informed consent. Participants were seated with spacing between desks (exam-style) and were instructed not to communicate with others. Within each session, participants were randomized 1:1 to the growth-mindset intervention or the active control using a computer-generated block randomization list. Allocation was concealed via pre-printed QR codes placed on the desks. Both participants and researchers were blind to condition, as all QR codes were uniformly formatted—printed in black and white on plain A4 paper—with no visible indicators to reveal allocation. Participants used their own phones to scan the QR code, which linked them to the online platform’s experimental program. All sessions were administered by the same primary researcher. Three research assistants supported logistics but remained outside the classroom once the session began. Participants were instructed to keep phones silenced and not to use other apps or external resources during the on-site, web-based component, and the primary researcher monitored compliance throughout the session.

Materials were displayed through the online experimental platform TC lab,^1^ a web-based system designed to administer experimental tasks and collect behavioral data online, including pre-test for growth mindset, the intervention, and the post-test for growth mindset. The entire web-based procedure took approximately 30 min, with the intervention lasting around 25 min. During the web-based component, participants progressed at their own pace; those who finished early waited quietly, and the subsequent logic-puzzle test began only after all participants completed the web-based component. To ensure task compliance, the online program incorporated predefined completion requirements based on pilot testing, and the experimenter reiterated task instructions during the session. Specifically, the reading materials in both conditions contained approximately 3,500 Chinese characters, and the program enforced page-level minimum time requirements that varied by page length (based on a minimum reading speed of ~400 characters per minute) before participants could proceed. The intervention also included three writing tasks with completion requirements (e.g., saying-is-believing: 5–10 sentences and ≥50 Chinese characters), and participants could not proceed until these requirements were met.

After the online tasks, the experimental paradigm from Malesky et al. (2022) was used to assess cheating behavior. The researcher sent pre-prepared logic puzzles to the participants, asking them to complete the test within 10 min. Participants completed the test on paper using a black gel pen; pencils and erasers were not provided. During the test, participants were explicitly instructed not to use their phones or any other external aids while completing the puzzles. Participants were informed that upon completing the test, they would be ranked based on their performance, with the first place receiving a first prize (RMB 30, approximately USD 4), the second and third places receiving second prizes (RMB 20, approximately USD 3), and no monetary prize for the remaining participants. This design was intended to simulate the awarding of scholarships in the university, thereby increasing the ecological validity of the experiment.

When 10 min had passed, a bell rang, and all participants were instructed to stop immediately, after which their test sheets were collected. The researcher was then interrupted by a planned and standardized phone call, enacted via a pre-programmed vibration reminder, and left the room. Outside the classroom, the researcher took photos of each participant’s original responses. Returning with both the test papers and the answer key, the researcher claimed to have an urgent matter to attend to and asked participants to grade their own answers using the answer sheet, under the pretense of avoiding delays. The researcher then exited the room for approximately 5 minutes. Participants graded their own responses, and upon return, the researcher collected the tests, ranked the scores, and distributed monetary rewards accordingly. Those who did not win a prize received a small gift (approximately worth RMB 2). No additional compensation was provided beyond the performance-based prizes and this small gift. To prevent answer leakage to students who had not yet participated, the researcher reminded participants not to share any information. Additionally, the test questions were presented in two different orders across experimental sessions, and all sessions were completed within 2 days to minimize the chance of answer sharing. After the study, the researcher compared the final submitted exams with the earlier photographed answers to identify changes to original responses, rather than simple scoring discrepancies. After completion of data collection, participants received a general debriefing regarding the purpose and procedure of the study. The debriefing plan was approved by the Institutional Review Board of the authors’ institution. To minimize potential risks and protect participants’ privacy, some deceptive elements were not disclosed at that time. The debriefing did not include individualized feedback or identification of any participant’s behavior. This procedure followed standard ethical guidelines for deception research.

**Figure 1:**
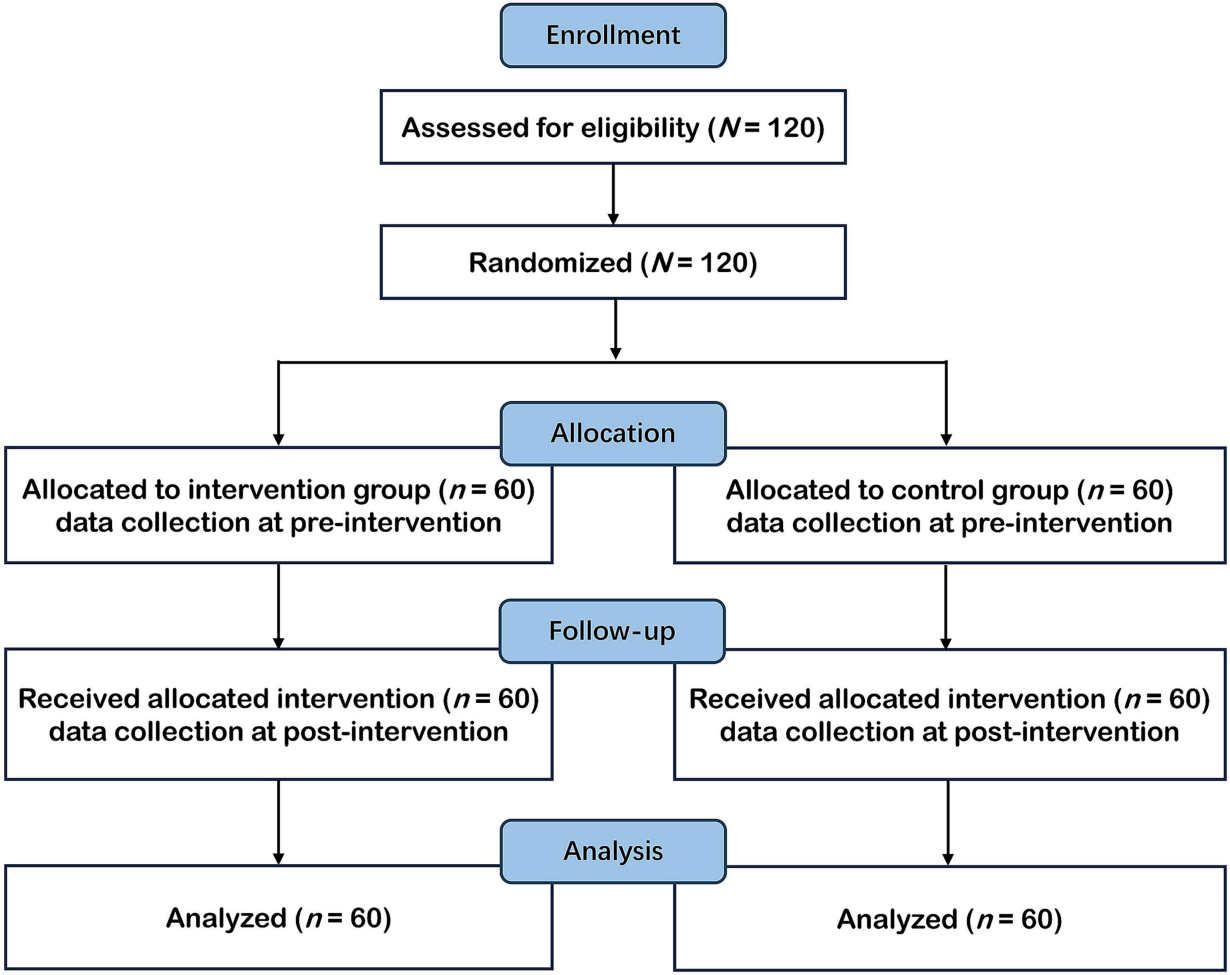
CONSORT flowchart.

All 120 participants completed the web-based intervention/control procedure, the logic-puzzle test, and the self-grading procedure without dropout, and no missing data were observed for these components. Therefore, all participants’ data were retained for the final analysis. Figure 1 presents the CONSORT flowchart.

#### 2.1.3 Intervention

The intervention involved a single self-guided online session lasting about 25 min. It drew on the refined version of the growth mindset intervention developed by Yeager et al. (2016), the protocol from Yeager et al. (2022), and Burnette et al.’s (2023) Framework for the Implementation of Mindset Interventions (FIMI). The program comprised four modules. In the first module, participants received a brief overview of the intervention framed in an indirect manner. They were told that the program was being developed for future college freshmen and their input was needed to improve it—an approach that fostered a sense of autonomy. This contrasts with a “direct” framing, where participants are explicitly told that the intervention is designed to benefit them. The second module provided scientific information about brain plasticity and the growth mindset of intelligence. It used a vivid metaphor— “the brain is like a muscle”— to illustrate that the brain becomes stronger through learning. Neuroscience findings were included to support the idea of malleable intelligence. Afterward, participants were asked to describe a personal experience of when they felt their brain had become stronger. The third module offered two actionable strategies to cultivate a growth mindset: engaging in challenging tasks and seeking genuine feedback from instructors and peers. In the final module, participants completed a “saying-is-believing” exercise, where they wrote a supportive letter to an incoming freshman facing academic challenges, thereby reinforcing and internalizing the concepts they had just learned.

The control group completed a structurally parallel online program consisting of four modules that were matched to the intervention in length, presentation format, and level of engagement. The first module was identical to that of the intervention group and provided a brief, indirectly framed introduction to the program. In the second module, control participants received general information about healthy brain use, focusing on basic principles of cognitive health rather than the malleability of intelligence. The third module introduced two practical strategies for maintaining brain health—adequate sleep and healthy nutrition. In the final module, control participants completed a writing exercise similar in structure to the intervention group’s “saying-is-believing” task, focusing on summarizing and conveying health-related information rather than reinforcing growth mindset beliefs.

#### 2.1.4 Measures

##### 2.1.4.1 Exam cheating

The logic test, adapted from Malesky et al. (2022), consisted of 10 questions targeting figural reasoning, verbal reasoning, and applied problem-solving. The task was of moderate difficulty, with an average of 6.22 correct responses (*SD* = 1.68) across the sample. As participants scored their own answers without monitoring, the difference between the experimenter’s recorded scores and participants’ self-reported scores was used as an indicator of cheating behavior. Cheating (binary) was coded as 1 if the self-reported correct count exceeded the photographed count, otherwise 0. Cheating magnitude (continuous) was computed as the self-reported minus photographed correct count.

##### 2.1.4.2 Growth mindset

Growth mindset was measured using the short version of the implicit theories of intelligence scale, which includes three items describing fixed mindset beliefs (e.g., “My intelligence is something about me that I cannot change very much.”) (Yeager et al., 2016). This short scale has been widely used in growth mindset research (Gunderson et al., 2018) and has demonstrated good reliability and validity in Chinese samples (Zhang et al., 2022). Responses were rated on a 6-point Likert scale ranging from 1 (“strongly disagree”) to 6 (“strongly agree”). All items were reverse-scored so that higher scores reflected a stronger growth mindset. In the present study, the Cronbach’s *α* coefficients for the pre- and post-intervention assessments were 0.87 and 0.90, respectively.

#### 2.1.5 Statistical analysis

All statistical analyses were performed using SPSS version 27.0. To evaluate intervention effects on growth mindset, a 2 (Group: intervention vs. control) × 2 (Time: pre vs. post) repeated measures ANOVA was employed. Simple effects were examined following significant interactions. For cheating outcomes, we used: (a) *χ*^2^ test for the binary cheating occurrence; and (b) independent-samples *t* test for the continuous cheating magnitude.

### 2.2 Results

#### 2.2.1 Manipulation check: growth mindset

There were significant main effects of time [*F* (1, 118) = 73.31, *p* < 0.001, partial *η*^2^ = 0.38] and group [*F* (1, 118) = 7.35, *p* < 0.01, partial *η*^2^ = 0.06], as well as a significant interaction effect between time and group on growth mindset [*F* (1, 118) = 39.48, *p* < 0.001, partial *η^2^* = 0.25]. Simple effects test indicated that no significant differences were found in growth mindset between students in the control and intervention groups at baseline, *F* (1, 118) = 0.001, *p* > 0.98. However, the intervention group reported a stronger growth mindset (*M* = 4.63, *SD* = 0.98) than the control group (*M* = 3.60, *SD* = 1.16) after intervention (see Table 2), *F* (1, 118) = 27.96, *p* < 0.001. The result indicated that after the online intervention, the growth mindset level of the participants in the intervention group was significantly improved, suggesting that the intervention of growth mindset was effective.

**Table 2:** Means and standard deviations of the variables.

Variables	Intervention group (*n* = 60)		Control group (*n* = 60)	
	*M*	*SD*	*M*	*SD*
GM T1	3.40	1.20	3.41	1.19
GM T2	4.63	0.98	3.60	1.16
Cheated (Yes/No)	0.15	0.36	0.30	0.46
Cheating scores	0.15	0.36	0.52	0.85

GM, growth mindset. T1 = pre-test, T2 = post-test. For the binary variable “Cheated” (Yes = 1, No = 0), the mean represents the proportion of participants who cheated.

#### 2.2.2 Cheating occurrence

Using the binary indicator of cheating (yes/no), the intervention group showed a lower proportion of cheating (15%) than the control group (30%), *χ*^2^ = 3.87, *p* < 0.05, *φ* = −0.18 (see Table 3).

**Table 3:** Chi-Square test results for group differences in cheating behavior.

Group	Cheated		*χ* ^2^	*p*
	Yes	No		
Intervention Group	9	51	3.87	< 0.05
Control Group	18	42		

#### 2.2.3 Cheating magnitude

When cheating magnitude was used as the outcome variable, a significant difference was observed between the experimental and control groups, *t (118)* = 3.07, *p* < 0.01, Cohen’s *d* = 0.56, 95% CI [0.19, 0.92]. Preliminary assumption checks indicated no substantial deviations from normality, supporting the use of the independent-samples *t*-test. As shown in Table 2, participants in the experimental group reported significantly lower cheating severity (*M* = 0.15, *SD* = 0.36) than those in the control group (*M* = 0.52, *SD* = 0.85), suggesting that growth mindset enhancement effectively reduced the extent of academic dishonesty.

Findings from Study 1 demonstrated that a single-session growth mindset intervention effectively increased students’ growth mindset levels. Furthermore, participants in the intervention group engaged in significantly less cheating than those in the control group on the subsequent test.

## 3 Study 2

Study 2 was designed as a follow-up to Study 1 to examine a possible mechanism linking growth mindset to exam cheating among university students. Based on the Study 1 finding that the growth mindset intervention reduced cheating, we expected growth mindset to be related to cheating and further tested how this relation might operate. Using a questionnaire-based approach, Study 2 measured growth mindset, the mindset meaning system, and self-reported exam cheating. We tested whether growth mindset was indirectly associated with cheating through the mindset meaning system.

### 3.1 Materials and methods

#### 3.1.1 Participants and procedures

A total of 489 undergraduate students were recruited from a university in Shandong Province, China. An anonymous, paper-based survey was administered in person in a group setting. The survey included demographic variables, growth mindset, mindset meaning system, and exam cheating, administered in that order. The questionnaire took approximately 5 min for participants to complete. Participants received a small stationery gift as compensation. After excluding 14 participants who failed attention checks or had excessive missing data, 475 valid responses were retained for analysis. The attention check was an embedded item instructing participants to select a specific response option; participants who selected any other option were excluded as failing the attention check. A sensitivity power analysis using G*Power (Faul et al., 2009) indicated that the final sample size (*N* = 475) was sufficient to detect an effect size as small as *r* = 0.16 with *α* = 0.05 (two-tailed) and power (1 − *β*) = 0.95.

Table 4 presents the demographic characteristics of the participants. The final sample included 475 students (74.53% female), aged 17 to 23 years (*M* = 19.62, *SD* = 0.80). Of these, 66 were first-year students, 267 were sophomores, and 142 were juniors. A total of 240 participants were classified as science-track majors, and 235 as arts-track majors, following the common major classification used in Chinese universities. In this sample, the science-track category included Mathematics, Chemistry, and Geography, and the arts-track category included History, Education, and Literature.

**Table 4:** General characteristics of the participants (*N* = 475).

Characteristic	Classification	*N* (%)
Gender	Male	121 (25.47%)
	Female	354 (74.53%)
Age	≤18	29 (6.11%)
	19	183 (38.53%)
	20	205 (43.16%)
	≥21	58 (12.21%)
Grade	1	66 (13.89%)
	2	267 (56.21%)
	3	142 (29.89%)
Major	Science-track	243 (51.16%)
	Arts-track	232 (48.84%)

#### 3.1.2 Measures

##### 3.1.2.1 Growth mindset

Growth mindset was measured using the same scale as in Study 1. In the current study, the Cronbach’s *α* coefficient for the scale was 0.92.

##### 3.1.2.2 Mindset meaning system

We assessed the mindset meaning system using the Brief Mindset Meaning System Indexes (Yeager and Dweck, 2020), which includes five single-item indicators: negative effort belief, performance-avoidance goal, challenge-seeking, helpless attribution, and resilient attribution. Each item was standardized (*z*-score), and an overall index was created by averaging the standardized scores. Higher scores indicate a stronger alignment with a fixed mindset.

##### 3.1.2.3 Exam cheating

Exam cheating was assessed using the exam cheating subscale from the Academic Dishonesty Questionnaire developed by Chen (2009). This subscale consists of 10 items describing specific cheating behaviors during exams (e.g., “Looking at a nearby classmate’s answer sheet during an exam”). Participants were asked to indicate how often they had engaged in each behavior since entering university. Responses were rated on a 5-point Likert scale ranging from 1 (“Never”) to 5 (“Very often”), with higher scores indicating more frequent engagement in exam cheating. In the present study, the Cronbach’s α coefficient for the scale was 0.84.

#### 3.1.3 Analytic strategy

First, we conducted missing data analysis and applied data imputation to address the missing values. Two participants were excluded due to excessive missing data: one did not provide demographic information, and the other had more than 50% missing responses. Little’s test for missing completely at random (MCAR) was nonsignificant, *χ*^2^ (12) = 14.62, *p* = 0.26, suggesting that the data were missing at random. Missing data (less than 1%) were imputed using the expectation–maximization (EM) algorithm. Second, descriptive statistics and bivariate correlations among the primary variables were computed. Third, we tested the indirect effect of growth mindset on cheating behavior through the mindset meaning system using Model 4 of the PROCESS macro (Hayes, 2013). Given that prior research has shown that demographic variables such as gender, age, and academic major may influence cheating behavior (Yu et al., 2017; Krou et al., 2021), they were included as covariates in the model. Indirect effects were tested using bias-corrected bootstrapping (5,000 resamples), with significance determined by 95% confidence intervals that did not contain zero.

### 3.2 Results

#### 3.2.1 Descriptive statistics and correlation of the variables

Table 5 presents the means, standard deviations, and correlations among the main study variables. The correlation between growth mindset and exam cheating was not statistically significant. However, growth mindset was negatively associated with the mindset meaning system, while the mindset meaning system was positively associated with exam cheating.

**Table 5:** Descriptive statistics and correlations for the main variables.

Variables	*M*	*SD*	1	2	3
1. GM	3.78	1.42	—		
2. MM	0.01	2.75	−0.41^***^	—	
3. Exam Cheating	1.33	0.42	−0.05	0.16^***^	—

^***^p < 0.001. GM, growth mindset; MM, the mindset meaning system.

#### 3.2.2 Testing the indirect pathway

**Figure 2:**
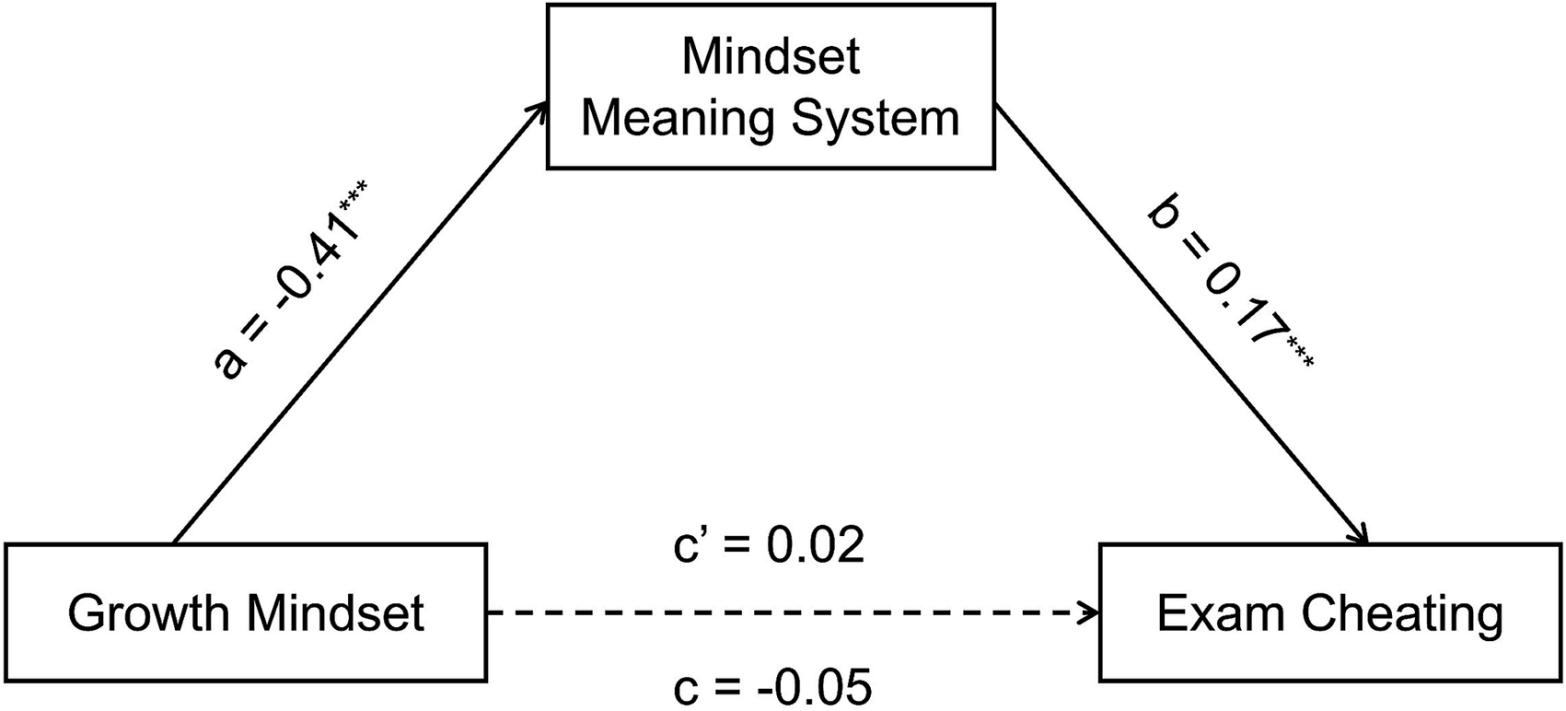
Results of the indirect-effect analysis. ^***^p < 0.001. The values presented in the figure are standardized coefficients.

After controlling for age, gender, and major, the total effect of growth mindset on exam cheating was nonsignificant [*β* = −0.05, *t* = −1.10, *p* = 0.27, 95% CI = (−0.14, 0.04)]. Growth mindset was negatively associated with the mindset meaning system [*β* = −0.41, *t* = −9.68, *p* < 0.001, 95% CI = (−1.37, −0.90)], and the mindset meaning system was positively associated with exam cheating [*β* = 0.17, *t* = 3.33, *p* < 0.001, 95% CI = (0.03, 0.10)]. Furthermore, the bias-corrected bootstrap analysis indicated a significant indirect effect of the mindset meaning system [indirect effect = −0.07, 95% CI = (−0.11, −0.03)]. Thus, although the total effect was nonsignificant, the results support a significant indirect pathway from growth mindset to exam cheating via the mindset meaning system (Preacher and Hayes, 2004). See Table 6 and Figure 2 for details.

**Table 6:** Testing the indirect effect of the mindset meaning system.

Predictor variables	Exam cheating		MM		Exam cheating	
	*β*	*t*	*β*	*t*	*β*	*t*
GM	−0.05	−1.10	−0.41	−9.68^***^	0.02	0.35
MM					0.17	3.33^***^
*R^2^*	0.01		0.17		0.32	
*F*	1.02		23.83^***^		3.05^*^	

^*^p < 0.05, ^***^p < 0.001. GM, growth mindset; MM, the mindset meaning system. Each column is a regression model that predicts the dependent variable at the top of the column.

## 4 Discussion

The present study aimed to examine the effectiveness of a growth mindset-based intervention in reducing cheating behaviors among university students and to explore its underlying mechanisms. The experimental findings of Study 1 showed that a brief, single-session growth mindset intervention significantly decreased cheating behaviors, offering causal evidence for the link between growth mindset and academic dishonesty. Study 2 further suggested that growth mindset was indirectly associated with cheating through the mindset meaning system. Together, these results provide empirical support for mindset-based interventions and highlight the broader applicability of mindset theory in promoting educational integrity.

### 4.1 Intervention effects on cheating behavior

In Study 1, the growth mindset intervention significantly enhanced university students’ growth mindset levels and reduced their exam cheating behavior, thereby supporting Hypothesis 1. Although prior cross-sectional studies (Thomas, 2017; Herdian and Rahayu, 2022) have documented negative correlations between growth mindset and academic dishonesty, experimental evidence of causality has been lacking. The present study addresses this gap by providing the first causal evidence, via a randomized controlled trial, for the effectiveness of growth mindset interventions in reducing cheating. This offers empirical support for mindset-based interventions targeting academic misconduct. When individuals believe in fixed innate abilities, they are more likely to experience anxiety and fear of failure, which can increase the likelihood of cheating. In contrast, growth mindset intervention emphasizes the malleability of abilities, thereby reducing performance-related anxiety and the temptation to cheat. These findings are theoretically aligned with the work of Mueller and Dweck (1998) and Zhao et al. (2017), which showed that praising intelligence may prompt students to cheat in order to preserve a positive self-image or reputation. Therefore, the current experimental results validate and extend existing knowledge by demonstrating that, growth mindset interventions can effectively diminish students’ likelihood of engaging in academic cheating.

Study 1 employed a single-session intervention, and the results showed that a brief, 25-min online session was sufficient to significantly enhance growth mindset levels among university students. This finding supports the effectiveness of the intervention protocol used in this study. Burnette et al. (2023) emphasized that the efficacy of growth mindset interventions depends largely on implementation fidelity. Our intervention was carefully designed to include core components identified as critical for mindset change (autonomy-supportive framing, “brain-as-a-muscle” metaphor, and reflective internalization strategies). The observed positive outcomes may therefore be attributed to this theoretically grounded design. In line with previous findings (Yeager et al., 2019; Schleider et al., 2020), our results further demonstrate that brief mindset interventions can reliably promote growth-oriented beliefs. Notably, this study expands the literature to include Chinese university students. Most prior growth mindset interventions have focused on Western populations, and there has been limited evidence regarding the plasticity of mindset in non-Western cultural contexts. In China’s highly competitive academic environment, where intelligence and achievement are heavily emphasized by both schools and families, fixed mindsets may be more easily fostered (Sun et al., 2021). Nevertheless, our findings suggest that even within such performance-oriented settings, growth mindset interventions remain effective, thus providing empirical support for the cross-cultural generalizability of mindset theory.

### 4.2 The mindset meaning system as a psychological mechanism

Study 2 extended the investigation by exploring the underlying mechanism linking growth mindset to cheating behavior, focusing on indirect pathway through the mindset meaning system. Findings indicated that growth mindset indirectly reduced cheating through shifts in students’ core motivational beliefs—including achievement goal orientations, beliefs about effort, and attributional styles. This sheds new light on how growth mindset can reduce academic dishonesty via specific psychological pathways. Although earlier studies have demonstrated correlations between growth mindset and academic misconduct (Thomas, 2017; Herdian and Rahayu, 2022), they have not examined the deeper structural mechanism underlying this link. The current study offers a distinct perspective by highlighting the mindset meaning system as a central explanatory framework. By connecting this system to established motivational models of cheating (Murdock and Anderman, 2006), our findings identify a theoretically coherent and empirically supported pathway through which mindset influences dishonest behavior. This expanded understanding contributes both to theoretical advancement and to the development of more targeted and practical interventions to reduce academic dishonesty.

Although growth mindset indirectly predicted exam cheating through the mindset meaning system, the direct correlation between growth mindset and exam cheating did not reach statistical significance. One possible explanation is that growth mindset may function as a distal factor compared to the more proximal motivational variables embedded in the meaning system. According to the Whitley’s (1998) model, proximal factors tend to have stronger associations with cheating behaviors than distal ones. This may explain why the mindset meaning system showed a significant correlation with cheating, while growth mindset itself did not. Another potential explanation is the reliance on self-reported measures of cheating behavior in Study 2, which are susceptible to social desirability bias. Participants may have underreported their cheating behavior (*M* = 1.33), particularly in a socially sensitive domain such as academic dishonesty. Additionally, the sample in Study 2 was predominantly female, and previous research has shown that female students tend to engage in less academic misconduct than their male counterparts. This gender imbalance may have contributed to the low levels of reported cheating, thereby weakening the observable association between growth mindset and exam cheating. Moreover, it is possible that different pathways linking growth mindset to cheating operate in inconsistent directions. Under such circumstances, a meaningful indirect pathway through the mindset meaning system may coexist with other pathways that do not uniformly discourage cheating, resulting in a weakened or nonsignificant direct association.

### 4.3 Limitations and future research

This study has several limitations that should be addressed in future research. First, Study 1 did not concurrently assess potential mechanisms; instead, these mechanisms were measured separately in Study 2. Although the current findings provide initial evidence for the effectiveness of a growth mindset intervention, our understanding of the psychological pathways through which the intervention reduces academic dishonesty remains limited. Future studies could incorporate the assessment of the proposed mechanisms into the experimental design or adopt longitudinal panels to examine temporal ordering. Second, although Study 1 employed an objective behavioral measure of cheating, which strengthens internal validity, the simulated nature of the task—a logic-puzzle activity with a relatively small monetary incentive—may not fully capture the pressures and motivations associated with cheating in real academic examinations. This limitation constrains the ecological validity of the findings. In addition, although an active control condition was used and matched to the intervention in structure and engagement, expectancy effects may not have been fully equivalent across conditions (Boot et al., 2013), which should be considered when interpreting the causal effects of the intervention. Future studies are encouraged to conduct field research or use more realistic exam simulations to better approximate authentic academic contexts. Third, the intervention implemented in this study was brief and single-session in nature. While significant effects were observed, such short-term interventions may not adequately capture the long-term influence of growth mindset on students’ beliefs and behaviors. Future research should examine the efficacy of extended or longitudinal growth mindset interventions. Fourth, the cross-sectional design of Study 2 limits causal inferences. Longitudinal studies are needed to confirm the long-term indirect effects of growth mindset on academic dishonesty through the mindset meaning system. Fifth, the Study 2 sample exhibited a notable gender imbalance, with female participants constituting 74.5% of the sample. Prior research suggests that male students tend to report higher levels of academic dishonesty, and this imbalance may therefore have attenuated the observed direct association between growth mindset and cheating. Accordingly, caution is warranted when generalizing the findings, particularly to male student populations. Future research should employ more gender-balanced samples and examine potential gender moderation effects. Lastly, the current sample consisted primarily of Chinese university students. While our findings support the intervention’s effectiveness in this context, it remains unclear whether these results generalize to students from different cultural backgrounds. Cultural factors may moderate the impact of growth mindset interventions. For example, studies have shown that performance orientation is more strongly associated with cheating in individualistic cultures, whereas learning orientation is linked to lower levels of cheating in collectivist cultures (Zhao et al., 2024a). Future research should explore how cultural context may shape the effectiveness of growth mindset interventions.

### 4.4 Practical implications

The findings of this study offer a novel practical approach to preventing academic cheating in higher education. In addition to traditional external regulatory strategies such as honor codes, proctoring systems, or punitive mechanisms, universities should also focus on cultivating students’ internal psychological resources, particularly the growth mindset. Strengthening students’ growth mindset may foster positive changes in key academic motivational factors—such as goal orientation, self-efficacy, and beliefs about effort—which in turn enhances their intrinsic commitment to academic integrity.

Study 2 showed that the mindset meaning system was more closely associated with cheating than growth mindset itself, highlighting the importance of proximal motivational meanings for understanding academic dishonesty. At the same time, directly changing specific motivational determinants (e.g., attributional tendencies, goal orientations) with brief and scalable interventions in educational practice may be challenging. In contrast, growth mindset interventions have been implemented in short, scalable formats and shown benefits in field settings, which makes growth mindset a practical upstream entry point. Future studies could develop scalable modules that more directly target key components of the mindset meaning system, and compare these approaches with growth-mindset interventions or integrate them to strengthen and sustain effects on academic integrity.

Furthermore, as highlighted by the “seed and soil” metaphor (Walton and Yeager, 2020), fostering a growth mindset should not be limited to individual-level interventions but requires a supportive educational environment. The effectiveness of such interventions depends on coordinated efforts across multiple levels, from instructors to institutions. For example, teachers should incorporate growth mindset principles in their pedagogy by emphasizing mastery-oriented goals, valuing the learning process over outcomes, and praising effort rather than innate ability. Institutions can also promote this mindset by shaping the learning environment—such as recognizing student improvement in scholarship criteria or integrating growth mindset concepts into course design. Embedding growth mindset principles across the educational ecosystem may help reduce academic dishonesty at its root and support students’ long-term development and psychological adjustment.

## 5 Conclusion

This research tested whether a brief growth-mindset intervention reduces exam cheating and whether the mindset meaning system explains this effect. Study 1 provided causal evidence that a single-session intervention increased growth-mindset beliefs and reduced both the likelihood and magnitude of cheating on a subsequent test. Study 2 further indicated that growth mindset was indirectly associated with lower cheating via a more adaptive mindset meaning system, whereas the direct association was nonsignificant. Taken together, these results add both causal and mechanistic support for mindset-based, scalable approaches to academic integrity.

## Data Availability

The raw data supporting the conclusions of this article will be made available by the authors, without undue reservation.

## Footnotes

1
https://www.testcloudlab.com/
